# Field-induced partial disorder in a Shastry-Sutherland lattice

**DOI:** 10.1038/s41467-023-39409-1

**Published:** 2023-06-19

**Authors:** Madalynn Marshall, Brianna R. Billingsley, Xiaojian Bai, Qianli Ma, Tai Kong, Huibo Cao

**Affiliations:** 1 Neutron Scattering Division, Oak Ridge National Laboratory, Oak Ridge, TN 37831 USA; 2 Department of Physics, University of Arizona, Tucson, AZ 85721 USA; 3 Department of Physics and Astronomy, University of Louisiana, Baton Rouge, LO 70803 USA; 4 Department of Chemistry and Biochemistry, University of Arizona, Tucson, AZ 85721 USA

**Keywords:** Phase transitions and critical phenomena, Magnetic properties and materials

## Abstract

A 2-Q antiferromagnetic order of the ferromagnetic dimers was found below *T*_N_ = 2.9 K in the Shastry-Sutherland lattice BaNd_2_ZnS_5_ by single crystal neutron diffraction. The magnetic order can be understood by the orthogonal arrangement of local Ising Nd spins, identified by polarized neutrons. A field was applied along [1 -1 0] to probe the observed metamagnetic transition in the magnetization measurement. The field decouples two magnetic sublattices corresponding to the propagation vectors **q**_**1**_ = (½, ½, 0) and **q**_**2**_ = (−½, ½, 0), respectively. Each sublattice shows a “stripe” order with a Néel-type arrangement in each single layer. The “stripe” order with **q**_**1**_ remains nearly intact up to 6 T, while the other one with **q**_**2**_ is suppressed at a critical field *H*_c_ ~1.7 T, indicating a partial disorder. The *H*_c_ varies with temperature and is manifested in the *H*-*T* phase diagram constructed by measuring the magnetization in BaNd_2_ZnS_5_.

## Abstract (web summary)

Shastry-Sutherland lattice materials offer a rich variety of accessible magnetic phases however, only a few have been observed to have ferromagnetic dimers, and among those, high quality single crystals are rare. Here, Marshall et al uses neutron diffraction on single crystals of BaNd2ZnS5, and show the existence of 2Q-antiferromagnetic order composed of ferromagnetic dimers and field-induced partial disorder. ‘

## Introduction

Exotic non-trivial magnetic behavior has emerged in magnetic systems with geometrical frustration. The kagome and triangular lattices are common examples of two-dimensional (2D) frustrated lattices^1,2^. More elusive is the 2D orthogonal dimer lattice famously realized in the material SrCu_2_(BO_3_)_2_^3,4^, which has been discovered to host a quantum spin liquid phase^5–8^. This lattice can be described by the Shastry-Sutherland (SS) model which consists of a 2D orthogonal arrangement of the spin dimers where the ratio between the two magnetic interactions, *δ* = *J*/*J’* with *J* and *J’* as the antiferromagnetic inter- and intra-dimer interactions, respectively, is critical for controlling magnetic states in the Shastry-Sutherland lattice (SSL)^9^. The *δ* ratio of 0.675 and 0.765 separate the dimer singlet, plaquette singlet and Néel phase, respectively, in the phase diagram of the SS model^10,11^.

Resultantly, rich magnetic phase diagrams have been constructed for SSL materials from the field-induced evolution of the magnetic order. One common feature are fractionalized magnetization plateaus, which likely originate from the transition of a dimer singlet ground state to the formation of superstructures of the field-induced triplet dimers such as in SrCu_2_(BO_3_)_2_^10,12–21^. The SSL is also found in families such as the rare earth tetraborides RB_4_ (R = rare earth)^22–24^, BaR_2_TO_5_ (R = rare earth, T = transition metal)^25–28^, R_2_T_2_In (R = rare earth, T = transition metal)^29–31^ and RE_2_Pt_2_Pb (R = rare earth)^32^. The magnetic ordered states can vary significantly from the insulator SrCu_2_(BO_3_)_2_ which possesses a Heisenberg type exchange interaction to the metallic RB_4_ family where a Ruderman-Kittel Kasuya-Yosida (RKKY) type interaction is observed between the moments giving it a long-rang order and possessing Ising-like moments oriented perpendicular to the SSL planes^33–35^. However, SSL materials such as Yb_2_Pt_2_Pb^32,36–39^, have been found to exhibit field-induced metamagnetic transitions associated with partially disordered states, where at low temperatures a Luttinger liquid state has been realized.

Consequently, only very few SSL materials were reported to possess ferromagnetic dimers, including the insulator BaNd_2_ZnO_5_^25^ and metallic TmB_4_^23,34,40^. Considering the newly explored BaR_2_TO_5_ family, although it is chemically diverse, the formation of the SSL will only occur for the lighter rare earth elements and successful single crystal growth has not been reported. The sulfide counterpart, BaR_2_TS_5_, which crystallizes into the same space group (*I*4/*mcm*) as the SSL BaR_2_TO_5_ materials, remains largely unexplored and recently large single crystals of BaNd_2_ZnS_5_^41,42^ have been synthesized exhibiting a *T*_N_ = 2.9 K. The dimers of the SSL in BaNd_2_ZnS_5_ are formed by the Nd atoms having inter-dimer lengths of 4.151 Å and intra-dimer lengths of 3.596 Å. A metamagnetic transition is observed in the *M*(*H*) with **H** along [1 −1 0], indicating this SSL material an excellent candidate for studying the intricate dimer physics from the field-induced magnetic phase evolution.

In this work, we report a 2-Q magnetic order in the SSL BaNd_2_ZnS_5_, determined by single crystal neutron diffraction. We utilize polarized neutrons to provide insight into the local magnetic anisotropy of the Nd spins and reveal the origin of the 2-Q magnetic order. The resulting field-induced evolution of the magnetic phases were characterized by magnetization measurements with the critical input from single crystal neutron diffraction. A partially disordered dimer liquid state was found and a “spin-flip-or-flop” mechanism was proposed to describe the dimer liquid state.

## Results and discussions

### Zero field magnetic structure

**Fig. 1:**
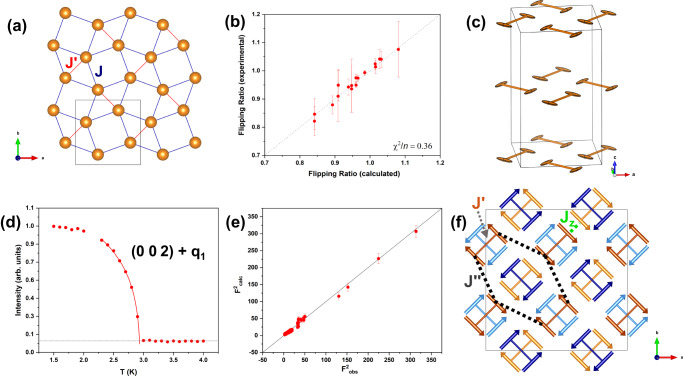
Zero-field magnetic structure refinement of BaNd_2_ZnS_5_. **a** SSL sublattice of Nd atoms in the *ab* plane*.* **b** Experimental versus calculated flipping ratio plot. **c** Local magnetic anisotropy of Nd dimers is showed by magnetic susceptibility tensors drew as ellipsoids in unit cell of BaNd_2_ZnS_5_. **d** Temperature-dependent order parameter of peak (½ ½ 2), red line is the empirical power law fitting, *I* ~ (*T*_m_ – *T*/*T*_m_)^2β^. **e** Experimental versus calculated structure factors at zero field. **f** 2-**Q** magnetic structure model for BaNd_2_ZnS_5_, the blue and orange represent the sublattices with **q**_**1**_= (½, ½, 0) and **q**_**2**_= (-½, ½, 0), respectively, and the light and dark color shades represent the different layers along the *c* axis. The *J’* and *J”* interaction paths correspond to the dimer bond and the dashed black line, respectively. The *J*_z_ represents the nearest spin–spin interaction between layers.

BaNd_2_ZnS_2_ exhibits 2D SSL layers of magnetic Nd^3+^ (*J* = 9/2) atoms separated by layers of Ba and Zn atoms and coordinated by S atoms. Nd^3+^ has a Kramers doublet ground state and behaves as a pseudospin 1/2^43^. Figure 1a, describes the Nd SSL where *J’* represents the intradimer interaction (nearest-neighbor) and *J* the interdimer interaction (next-nearest-neighbor), a typical SS interaction model. To understand the magnetic anisotropy of the Nd^3+^ spins in BaNd_2_ZnS_5_, we measured the local magnetic susceptibility tensor of the Nd^3+^ spins by polarized neutron diffraction^44,45^. The local symmetry of the Nd atomic site, the 8*h* site of space group *I*4/*mcm*, implies the principal axes of the ellipsoid are along the [1 1 0], [1 −1 0] and [0 0 1] directions. The bulk magnetic measurements have revealed the magnetic moments are easy in-plane^42^. Therefore, only one-field-direction along [1 −1 0], was selected to detect the in-plane magnetic anisotropy of the Nd^3+^ spins. By measuring reflections in spin-up and spin-down neutron channels, we obtained 17 good-quality flipping ratios to refine two free susceptibility tensor parameters in-plane. A suitable fitting of the flipping ratios could be reached, as shown by the Fig. 1b plot of the experimental versus calculated flipping ratios, using the software CrysPy. The in-plane principal axes of the Nd^3+^ magnetization ellipsoids, Fig. 1c, were found to be orthogonal to the dimer bond consistent with an Ising-spin nature with lengths *χ*_//_ = 0.183(22) μ_B_/T and *χ*_┴_ = 0.033(22) μ_B_/T. Similar to Yb_2_Pt_2_Pb^32^ the magnetic moments are found within the plane of the SSL and orthogonally arranged between two magnetic sublattices that satisfy the Ising behavior, contrary to that in SrCu_2_(BO_3_)_2_^14^ and TmB_4_^23^. Instead of the well-known SSL interaction model, the resulting formation seemingly favors an effective square lattice magnetic model where *J’* and *J”* are the potential interaction paths, as indicated by the orange line (i.e. the dimer bond) and the dashed black line, respectively, in Fig. 1f^32^. The symmetric terms of interaction *J* produce zero energy contribution due to the orthogonality of the spin arrangement between the neighboring orthogonal dimer bonds (shown in Fig. 1a as a standard SS spin model but ignored in Fig. 1f due to the zero-energy contribution from *J* for the Ising spins). While the antisymmetric terms of *J* in the spin Hamiltonian (see the full description in the SI) are likely weak as well, this will be shown by the field measurements presented later. Future inelastic neutron scattering measurements are needed to further confirm the interaction speculation here and interpret the spin dynamics in BaNd_2_ZnS_5_.

From the temperature-dependence of the magnetic scattering at (½ ½ 2), Fig. 1d, the magnetic order appears at ~ 3 K, consistent with the reported *T*_N_ = 2.9 K from the magnetic susceptibility measurements^42^. The solid red line in Fig. 1d, corresponds to the power law fitting of the intensity, *I* ~ (*T*_N_ – *T*/*T*_N_)^2β^, with a *T*_N_ reasonably fixed at 2.95 K, and a *β* ~ 0.08(1) which is smaller than the expected *β* = 1/8 for a 2D Ising system^32^ and could be a result of the nature of the spin dimer lattice. As a product of the single crystal polarized neutron diffraction results, a suitable magnetic structure model could be immediately determined since the fit could be appropriately constrained with moments perpendicular to the dimer bonds. A 2-**Q** AFM model consisting of two magnetic sublattices indexed by the propagation vectors **q**_**1**_ = (½, ½, 0) and **q**_**2**_ = (-½, ½, 0), resembling the AFM 2-**Q** structure for BaNd_2_ZnO_5_^25^ but with a different magnetic symmetry, best fit the zero-field data. The magnetic symmetry *P*_*C*4/*nnc* (#126.385) was then determined using the k-SUBGROUPSMAG program from the Bilbao Crystallographic server. Based on the body centered symmetry, the SSL layers are separated by a centering translation resulting in two inequivalent propagation vectors that each connects to one magnetic sublattice with a “stripe” order when viewing two layers together. Therefore, a FM interlayer interaction, *J*_z_, also needs to be considered, which is likely as weak as *J”* due to the larger atomic distance between interacting spins, however, necessary to stabilize the magnetic order at zero field, distinct from the magnetic order reported in BaNd_2_ZnO_5_^25^. Each SSL layer individually exhibits a Néel phase arrangement where the potential interaction paths consist of a dominant FM *J’* and a weak AFM *J”*. The refined Nd magnetic moment was determined to be 2.6(1) μ_B_. Figure 1e shows a plot of the calculated structure factor square (*F*^2^_calc_) versus the observed one (*F*^2^_obs_) and the magnetic structure can be seen in Fig. 1f where the orange and blue atoms represent the two different sublattices and the overlapping layers along the *c* axis are indicated by the light and dark color shades.

### Field-induced phase evolution

**Fig. 2:**
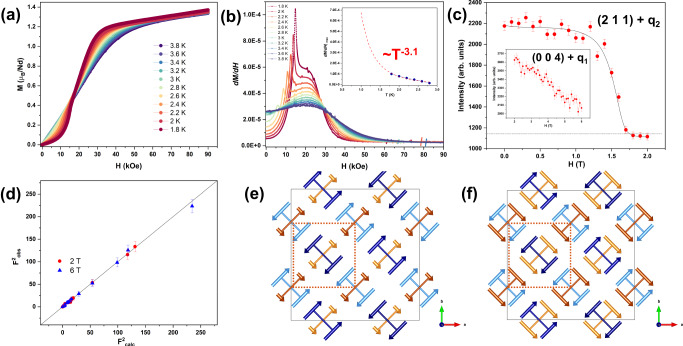
Field induced phase evolution and magnetic structure refinement at 2 and 6 T. **a** Magnetization measurement with **H**//[1 −1 0] from 1.8 to 3.8 K. **b** Plots of d*M*/d*H* measured at constant temperature from 1.8 to 3.8 K, data were smoothed for the derivation, with an inset of d*M*/d*H*|*max* versus temperature as derived from the maximum value of the sharp peak in d*M*/d*H*, the red line represents the power-law dependence ~ *T*^−n^ (*n* = 3.1). **c** Field-dependent order parameter from 0 to 2 T at (1.5 1.5 1). The inset for the field-dependent order parameter from 2 to 6 T at (½ ½ 4). **d** Experimental versus calculated structure factors at 2 T (red circles) and 6 T (blue triangles). **e** The refined magnetic structure of the partially disordered phase at 2 T and **f** the field polarized state at 6 T with the square lattice of FM spin dimers shown by the dashed dark orange lines.

The magnetization curves below *T*_N_ of BaNd_2_ZnS_5_ show kinks around 1.7 T for fields along the [1 −1 0] direction, as shown by Fig. 2a, indicating a metamagnetic transition. To investigate this transition, single crystal neutron diffraction measurements were performed with an applied magnetic field of 2 and 6 T parallel to the [1 −1 0] direction. As the field-induced transition emerges around 1.7 T, at 1.4 K, the field-dependent magnetic scattering at (1.5 1.5 1) disappears at 1.7 T (see Fig. 2c) signifying the stripe phase with **q**_**2**_ = (-½, ½, 0) is no longer present. Therefore, the kink shown in the magnetization measurement is a signature of the magnetic order-disorder transition in the magnetic sublattice with spins parallel to the field and the corresponding field can be viewed as the critical field *H*_C_ for this transition. For the other magnetic sublattice, the magnetic peak (½ ½ 4) gradually decreases with the field increasing but the majority of the magnetic peak signal is maintained up to 6 T (see Fig. 2c inset). By analyzing the neutron diffraction data collected at 2 and 6 T at 1.4 K, the refined magnetic moments for the stripe phase of the **q**_**1**_ magnetic sublattice with spins along [1 1 0], perpendicular to the field direction, were determined to be 2.8(1) and 2.6(1) μ_B_, respectively.

At 2 and 6 T the observed magnetic reflections from the diffraction patterns could be all indexed by **q**_**1**_ = (½, ½, 0), while no peaks could be indexed with **q**_**2**_ = (−½, ½, 0) when considering the body centering translation symmetry. Note, the body-centered unit cell is not a primitive cell and so **q**_**1**_ and **q**_**2**_ are not equivalent. From the k-SUBGROUPSMAG program, the low symmetry space group *P*1 (#1.1) was initially selected to test the potential magnetic models under field. The resulting refinement reveals a partially disordered state of ferromagnetic dimers at 2 T in one magnetic sublattice (Fig. 2e) and while the AFM order in the other magnetic sublattice with moments along [1 1 0] (**q**_**1**_ magnetic sublattice) survives (Fig. 2e and 2f). The results also indicate the two magnetic sublattices are interaction-decoupled and can be separated under an applied field, which confirms that the interaction *J* between the two magnetic sublattices is weak, i.e., no strong antisymmetric exchange interactions between the orthogonally arranged neighboring Nd spin dimers. This separation can also be observed as two *T*_N_’s connected to the two sublattices under field, see Supplementary Figs. S1 and S2. Considering the magnetic interaction distance and the localized *f*-electron feature for the rare-earth spins, both *J”* and *J*_z_ are much weaker compared to the intra-dimer interaction *J’*. Therefore, the SSL in BaNd_2_ZnS_5_ can be viewed as two decoupled square lattices of ferromagnetic dimers that are loosely 3-dimensionally connected. While the magnetic phase transitions induced by the field up to 6 T along [1 −1 0] at 1.4 K is likely only within the **q**_2_ magnetic sublattice with Nd^3+^ moments along [1 −1 0]. Therefore, the following discussions will be focused on only the magnetic dimer square sublattice with spins parallel to the field [1 −1 0] (the square lattice of FM spin dimers is shown in Fig. 2e and f as the dark orange line for the magnetic sublattice with spins along [1 −1 0]).

**Fig. 3:**
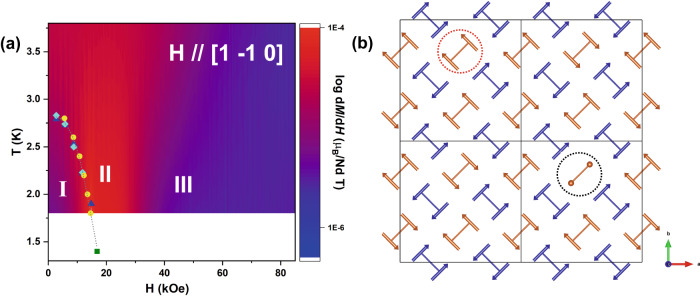
A constructed phase diagram along with possible dynamical magnetic patterns for disordered state. **a** Field versus *T*_N_ (*H*–*T*) phase diagram with **H**//[1 −1 0], the magnetic susceptibility, magnetization and neutron data are represented by the light blue diamonds and blue triangles for the two measurements which were performed, yellow circles and green squares, respectively. The *H*-*T* phase diagram overlays a contour plot mapping the values of d*M*/d*H* obtained at constant temperatures, the values of d*M*/d*H* are represented by the color scale. **b** A possible dynamical magnetic pattern at the *H*_C_, the dotted red-circle highlights the spin-flip transition from *S*_*Z*_ = −1 to *S*_*Z*_ = +1 and the dotted black-circle highlights the spin-flop transition from *S*_*Z*_ = −1 to *S*_*Z*_ = 0, the orange balls in the black-circle symbolize no magnetic moment is present.

Neutron diffraction revealed that the **q**_2_ stripe order is fully suppressed by the field at the critical field *H*_C_ ~ 1.7 T. Field-induced magnetic signal on top of the nuclear Bragg peaks were refined as uniformly aligned moments of 1.2(2) μ_B_ at 2 T for the magnetic sublattice with the spin Ising axis along [1 −1 0] // **H**, i.e., 0.6(1) μ_B_ per Nd^3+^ if averaging it for the whole magnetic lattice, consistent with the increased magnetization in the bulk measurement. If we consider the model of the square lattice of ferromagnetic dimers as described above, each ferromagnetic dimer includes two parallel aligned spin-half moments and so makes a spin-1 dimer with *S* = 1 as the ground state. When a field is applied, two kinds of dimer spin transitions among three magnetic components (*S*_Z_ = −1, 0, +1) can occur and cause the order-disorder transition and yield the average induced moment as seen by neutrons. One is a spin-flip transition from *S*_Z_ = −1 to *S*_Z_ = +1 and the other one is a spin-flop transition from *S*_Z_ = −1 to *S*_Z_ = 0, the quantum version of the well-known spin-flop transition in a weak-magnetic-anisotropic AFM system^46,47^. Both spin component transitions are illustrated in Fig. 3b. The flipped or flopped spin dimers are disordered in the lattice space because no additional superlattice ordering peaks were observed. Therefore, we refer to this partial disorder as a liquid-like state, i.e., dimer liquid. More dynamic measurements are needed to further characterize the nature of the dimer liquid state in contrast to the reported Luttinger liquid state in the SSL material Yb_2_Pt_2_Pb^48^, the quantum spin liquid phase in the Kitaev honeycomb-lattice RuCl_3_^49^, and the magnetization plateau phases in the SSL material SrCu_2_(BO_3_)_2_^10,12–21^. At 6 T the square magnetic sublattice with Nd spins along [1 −1 0] enters a field polarized state and has a refined magnetic moment of 2.8(1) μ_B_, the magnetic structure is shown in Fig. 2f.

### Phase diagram and spin dimer liquid

We can construct the field-temperature (*H*–*T*) phase diagram from the magnetization data upon varying temperature since neutron diffraction reveals that the kink observed in the magnetization indicates the disorder transition from the stripe order in the **q**_2_ magnetic sublattice. The critical field *H*_C_ at each temperature can be better observed as a sharp peak in the plots of d*M*/d*H* (see Fig. 2b), where upon cooling, the *H*_C_ shifts towards higher fields, *H*_C_ ~1.5 T at 1.8 K. Above *H*_C_, the transition to the field polarized state can be seen by the broad bump feature at higher field. A contour plot mapping the values of d*M*/d*H* obtained from 1.8 to 3.8 K, is depicted in a *H* versus *T* (*H*–*T*) phase diagram describing the magnetic sublattice with spins parallel to the field direction [1 −1 0], Fig. 3a, based on the bulk magnetization (circles) and susceptibility (diamonds and triangles) measurements under the field along [1 −1 0] and neutron data (square). Phase I represents the 2-**Q** magnetically ordered stripe phase while phase II is the dimer liquid phase. The contour plot of d*M*/d*H* clearly defines the regions for Phase I and II and furthermore the transition to the field polarized state as Phase III. According to the neutron diffraction measurement at 1.4 K, no additional magnetic order was observed when the stripe phase enters the spin dimer liquid state, Phase II, at 1.7 T, indicating the critical region is further narrowed towards a possible critical point.

A similar temperature dependence of the behavior of *H*_C_ in Fig. 3a has been observed in the phase diagrams of geometrically frustrated lattices exhibiting field-induced quantum criticality, such as CoNb_2_O_6_^50,51^ and RuCl_3_^49^, constructed from heat capacity measurements under field. A possible explanation of such a *H*–*T* behavior in BaNd_2_ZnS_5_ can be explained by field-melting the stripe ordered phase through “spin-flip-or-flop” transitions. The *H*–*T* phase diagram could also suggest the possibility that quantum criticality may exist in BaNd_2_ZnS_5_ at the lowest temperature. Additional evidence is shown by the plot of d*M*/d*H*|*max* versus *T* in the Fig. 2b inset that demonstrates the power law fitting ~*T*^-n^, where it has been constrained to *n* = 3.1 to assess the possibility of quantum critical fluctuations as determined for the antiferromagnet CePtIn_4_^52^. Down to 1.8 K BaNd_2_ZnS_5_ resembles that of the linear higher temperature region in the power law fitting of CePtIn_4_, suggesting a certain similarity of the two systems. To explore these scenarios and reveal the enigmatic magnetic ground state of BaNd_2_ZnS_5_, low temperature heat capacity, magnetization and inelastic neutron scattering measurements are required. In summary, single crystals of BaNd_2_ZnS_5_ from the BaR_2_ZnS_5_ family have been successfully synthesized and studied by magnetic bulk measurement and neutron diffraction under magnetic field. Ising magnetic anisotropy of the Nd spins is revealed by the local magnetic susceptibility method with polarized neutrons and their Ising directions are orthogonal to the dimer bonds. Such an arrangement of Ising spins implies symmetric exchange interactions cannot couple the orthogonally arranged dimers. The zero-field magnetic order is a 2-**Q** AFM order of FM dimers with Nd spins along their local Ising directions. Two magnetic sublattices with **q**_**1**_ = (½, ½, 0) and **q**_**2**_ = (-½, ½, 0) constitute the 2-**Q** structure and respond to the field along [1 −1 0] differently. The **q**_**2**_ magnetic order is suppressed at a critical field, responsible for the kink observed in the magnetization measurement. While the **q**_**1**_ magnetic sublattice stays mostly unchanged until 6 T. According to this information, we built the *H*-*T* phase diagram from the bulk magnetization and magnetic susceptibility. A critical region was manifested as a spin dimer liquid phase growing out between the stripe phase at lower field and the field polarized phase at upper field. “Spin-flip” and “spin-flop” mechanisms were proposed to explain the formation of the liquid state. Whether the dimer liquid phase could condense at an ultra-low temperature deserves further study. As BaNd_2_ZnS_5_ exhibits a unique 2-**Q** magnetic square lattice with weak interactions between orthogonal dimers, it can be an exciting candidate to exhibit unique high order symmetries and a potential host for exotic quantum phases. Future dynamic studies including inelastic neutron scattering can provide insight into magnetic interactions and emergent states in the BaNd_2_ZnS_5_ SSL and discover interesting dynamic properties of ferromagnetic dimers. An effort in synthesizing large, high quality single crystals is thus called for, as available high-quality single crystals make all these possible.

## Methods

### Neutron diffraction

To determine the magnetic order single crystal neutron diffraction experiments on the HB-3A DEMAND^53^ at the High Flux Isotope Reactor at Oak Ridge National Laboratory. A 1.5 mm sample was measured with the two-axis mode down to 1.4 K using a cryomagnet and a wavelength of 1.542 Å from a bent Si-220 monochromator^54^. The measurement was performed with an applied magnetic field of 0, 2 and 6 T parallel to [1 −1 0]. The Bilbao Crystallography Server^55^ was used for the magnetic symmetry analysis and Fullprof software^56^ for the magnetic structure refinement. Polarized single crystal neutron diffraction measurements was likewise performed on the HB-3A DEMAND with a polarized neutron beam of 1.542 Å and the calibrated neutron polarization is 72%. The crystal was loaded in a closed-cycle refrigerator with a permanent magnet set providing the fixed field of 0.5 T along [1 −1 0]. We measured 17 flipping ratios at 5 K, above *T*_N_. To analyze the resulting flipping ratios the CrysPy software^57^ was used.

## Supplementary information

Supplementary Information

## Data Availability

All relevant data are available from the corresponding author upon reasonable request.
