# Supplementary material for: Field-induced partial disorder in a Shastry-Sutherland lattice
Source: Nat Commun. 2023 Jun 19;14:3641. doi: 10.1038/s41467-023-39409-1 (PMC10279730; doi:10.1038/s41467-023-39409-1)
Supplement: Supplementary file 1 — Supplementary Information [file 41467_2023_39409_MOESM1_ESM.pdf]

Supplementary Information

Field-Induced Partial Disorder in a Shastry-Sutherland Lattice

Madalynn Marshall[1], Brianna R. Billingsley[2], Xiaojian Bai[1,4], Qianli Ma[1], Tai Kong[2,3], Huibo Cao[1*]

[1]*Neutron Scattering Division, Oak Ridge National Laboratory, Oak Ridge, Tennessee 37831, USA*

[2]*Department of Physics, University of Arizona, Tucson, Arizona, 85721*

[3]*Department of Chemistry and Biochemistry, University of Arizona, Tucson, Arizona, 85721*

[4]*Department of Physics and Astronomy, University of Louisiana, Baton Rouge, Louisiana, 70803*

**email:* caoh@ornl.gov

1. Spin Hamiltonian

By considering J - the nearest orthogonal spin interactions, J' - intradimer spin interactions, J'' - the 2nd nearest interdimer interactions, J_z – the nearest interlayer spin interactions (see Figure 1a and 1f in the main manuscript), the spin Hamiltonian in the SSL $BaNd_2ZnS_5$ is expressed as,

$$H = \sum_{nn'} S_n \cdot J \cdot S_{n'} + \sum_{ii'} S_i \cdot J' \cdot S_{i'} + \sum_{jj'} S_j \cdot J'' \cdot S_{j'} + \sum_{kk'} S_k \cdot J_z \cdot S_{k'}$$

According to the bond symmetry analysis using Su(n)ny [1], the interaction tensors are listed as

$$J = \begin{bmatrix} X & Z+P & 0 \\ Z-P & X & 0 \\ 0 & 0 & Y \end{bmatrix}, J' = \begin{bmatrix} A & D & 0 \\ D & B & 0 \\ 0 & 0 & C \end{bmatrix}, J'' = \begin{bmatrix} E & H & 0 \\ H & F & 0 \\ 0 & 0 & G \end{bmatrix},$$

$$J_z = \begin{bmatrix} I & K+L & M-N \\ K-L & I & -M-N \\ M+N & -M+N & R \end{bmatrix}.$$

and 18 non-zero elements represent the allowed interactions.

As discussed in the "*Field-Induced Phase Evolution*" section in the main manuscript, the neutron diffraction data suggest the FM interaction J' is dominant and the inter-dimer interaction J is weak, which decouples two magnetic sublattices as shown by the field-dependence of magnetic orders. While $J''$and J_z interactions in each magnetic sublattice are required to stabilize the observed

magnetic order although they are not expected to be strong due to the larger bond distances. This leaves the effective Hamiltonian to be $H = \sum_{ii'} S_i \cdot J' \cdot S_{i'} + \sum_{jj'} S_j \cdot J'' \cdot S_{j'} + \sum_{kk'} S_k \cdot J_z \cdot S_{k'}$.

Within the context of the main manuscript, we discussed the interactions using the "symmetric" exchange (*X,Y,Z; A,B,C,D; E,F,G,H; I,R,K,M*) and "antisymmetric" exchange (*P*, *L*, and *N*) in the matrix representation of J, J', J'' and J_z and evaluated the relative interaction strengths according to the diffraction data. To quantitatively determine the interaction matrix of the spin Hamiltonian, one needs to measure spin dynamics by inelastic neutron scattering, which requires larger crystals.

2. Field-dependent specific heat and T_N

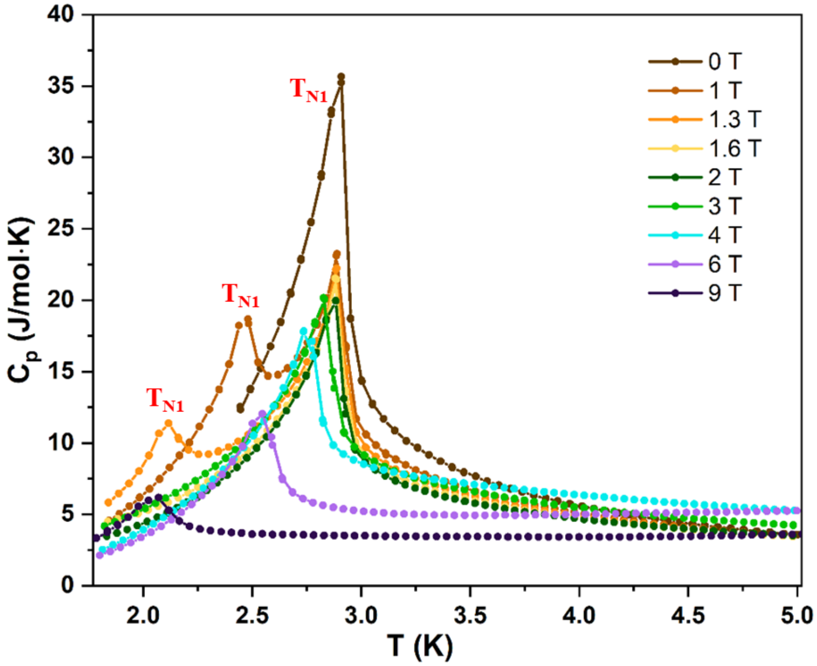

Supplementary Figure S1. Bulk temperature dependent specific heat measurements down to 1.8 K for a series of fields from 0 – 9 T. Measurements were performed on a Quantum Design physical property measurement system (Dynacool) using the two-tau relaxation method on a 2.7mg sample with **H** // (1 1 0). As indicated by peaks in each specific plot, one magnetic transition splits into two under field. The transition associated with the magnetic sublattice with spins parallel to the field [1 -1 0] is labeled as T_{N1}. T_{N2} is for the other one with spins perpendicular to **H**.

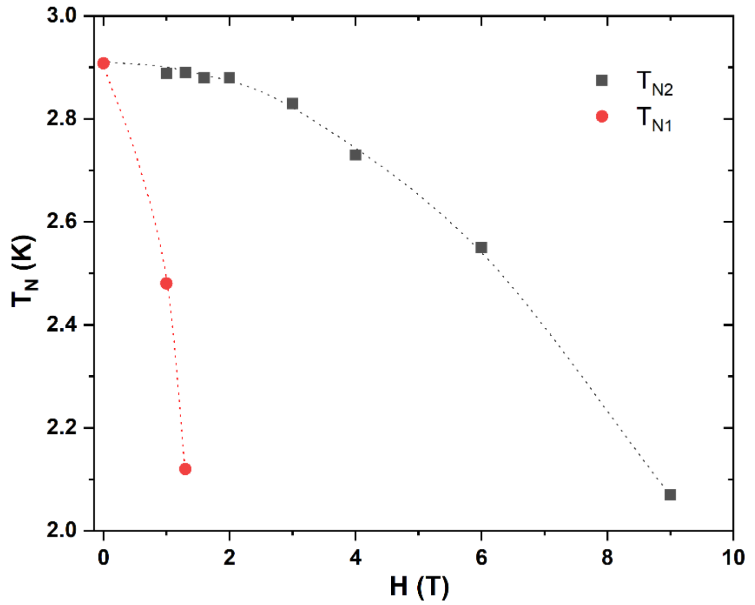

Supplementary Figure S2. Transition temperature (T_N) versus field **H** // (1 1 0). The data are extracted from the bulk temperature dependent specific heat measurements for a series of fields from 0 – 9 T as shown in Figure S1.

3. Magnetization without hysteresis

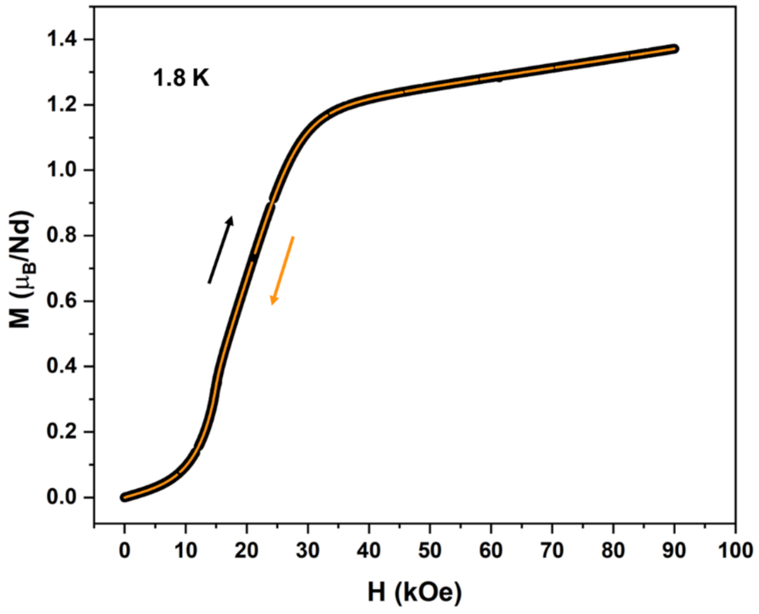

Supplementary Figure S3. Magnetization measurement shows no hysteresis observed at 1.8 K, the black and orange line represents the field wrapping up and down as indicated by the arrows.

References

1. Barros, Kipton, Matin, Sakib, Li, Ying Wai, and Wilson, Matthew. Contributing to Sunny open-source code. Computer Software. https://github.com/SunnySuite/Sunny.jl. USDOE Office of Science (SC), Basic Energy Sciences (BES). 01 Jul. 2022. Web. doi:10.11578/dc.20220715.3.
